# 

**Published:** 1832-04-01

**Authors:** 


### BIBLIOGRAPHICAL RECORD.

1. The Cyclopædia of Practical Medicine. Edited by Drs. FORBES, TWEEDIE, and CONOLLY. Part I. pp. 112, royal 8vo, double columns. January 1, 1832. To be continued monthly. Abdomen to Aueurism. Price 5s. each Part.

2. Thoughts on the Best Means of Lessening the Destructive Progress of Cholera. By JOSHUA BROOKES, F.R.S. &c. 8vo, pp. 15. January, 1832.

3. Die Cholera in Berlin, &c. By Dr. GODFRED REICH. 8vo, pp. 146. Berlin, 1831.

☞ *Reported on in this Number.*

4. Ueber das Naturliche System der Practeschen Medicin. VON L. W. SACHS. pp. 82.

5. Rencension der Annales Scholæ Clinicæ Ticinenses, &c. By PROFESSOR HILDENBRAND. 1830.

6. Die Grundlage der Heilkunde. By Dr. G. C. REICH, Professor of Medicine, &c. 8vo. pp. 298.

7. History of the Chronic Phlegmasiæ, or Inflammations, founded on Clinical Experience and Pathological Anatomy; exhibiting a view of the different varieties and complications of these diseases, with their various methods of treatment. By F. S. V. BROUSSAIS, M.D. &c. translated from the fourth French edition, by Dr. J. HAYS, and R. E. GRIFFITH, M.D. of Philadelphia. Two volumes, 8vo. Longman and Co. London, 1831.

☞ *We advise all those who have not the original French work to avail themselves of this opportunity to peruse Broussais in the English language.*

8. Observations on the Origin and Treatment of Cholera and other Pestilential Diseases, and on the Gaseous Oxide of Nitrogen as a Remedy in such Diseases, &c. By JOHN HANCOCK, M.D. Med. Bot. and Zoolog. Soc. &c. 8vo. pp. 64. 1831.

☞ *We neglected to record this in our last number.*

9. Hints on the Constitution of Dispensaries, &c. By JOHN STORER, M.D. F.R.S. Consulting Physician to the General Hospital near Nottingham. 8vo, pp. 92. 1832.

10. The Anatomy of Drunkenness, By ROBERT MACNISH, Author of the Philosophy of Sleep, &c. Fourth Edition, small 8vo. pp. 266. M'Phun, Glasgow, January, 1832.

☞ *The 4th Edition of a work which one would expect the drunkard to fear, and the sober man to consider as inapplicable to his own case, is a strong proof that the book has attractions for both classes. The present Edition has been very materially enlarged by a variety of new and useful information. It will do more good than all the temperance-societies in Great Britain.*

11. On the Portable Sudatory, or Hot Air-Bath, &c. By M. LA BEAUME, Medical Galvanist, &c. 8vo, pp. 84. January, 1832.

☞ *M. Le Beaume has very much improved the Portable Hot Air-Bath.*

12. On the Phenomena of Dreams. By WALTER C. DENDY, M.R.C.S. &c. Duodecimo, pp. 154. January, 1832.

☞ *This is one of the most amusing and scientific investigations of the subject which we have yet seen.*

---

13. Memoir sur le Cholera Morbus de l'Inde. Par P. F. KERAUDREN, M.D. Octavo, pp. 39. Bailliere, 1832.

☞ *We recommend this pamphlet.*

---

14. Address to the Members of the Hunterian Medical Society (Ed.) Delivered Nov. 6th, 1831. By NATHANIEL ROGERS, Senior President.

---

15. Principles and Practice of Obstetric Medicine, &c. By D. D. DAVIS, M.D. Part III. pp. 64, with two plates. January, 1831. *See Review.*

---

16. Remarks on the Anatomy Bill now before Parliament, in a Letter to the Right Honorable Lord Althorp. By G. J. GUTHRIE, F.R.S. 8vo, pp. 8, sewed. Price 6d. Sams, St. James's Street, Jan. 1832.

---

17. Letters on the Cholera. By WHITELAW AINSLIE, M.D. &c. 8vo, pp. 44, sewed. Wilson, Princes Street. 1832.

---

18. The Modern Anatomy of the Human Spleen; with Strictures on Sir A. Carlisle's Theory, &c. By P. MAC NOLTY, Surgeon, &c. 8vo. Renshaw and Rush, 1832.

---

19. Cholera, its Character and Treatment; with Remarks on the Identity of the Indian and English, and a particular reference to the Disease as now existent at Newcastle and neighbourhood. By CHARLES TURNER THACKRAH. 8vo. pp. 60. Leeds, January, 1832.

---

20. Remarks on the History and Treatment of Delirium Tremens (from the Transactions of the Massachuset's Medical Society.) By JOHN WARE, M.D. 8vo. 1831.

---

21. A Series of Experiments Performed for the purpose of shewing that Arteries may be Obliterated without Ligature, Compression, or the Knife. By BENJAMIN PHILLIPS. 8vo. stitched. pp. 66. London, 1832.

22. A Treatise on Physiology applied to Pathology. By F. S. V. BROUSSAIS, M.D. Translated from the French. By JOHN BELL, M.D. and R. LA ROCHE, M.D. Third American Edition, with Notes and copious Index. 8vo. pp. 666, Carey and Lea, Philadelphia, 1832.

☞ *This is a very valuable volume, and well deserves importation into this country.*

---

23. A Letter addressed to the Lord Chancellor on the Study of Anatomy. By JAMES C. SOMERVILLE, M.D. 8vo, pp. 15. January, 1832.

---

24. Letters on the Cholera Morbus, shewing that it is a Non-Contagious Disease. By a Professional Man of thirty years' experience. 8vo, pp. 56.

☞ *These Letters are a republication (with additions, &c.) of the talented letters, signed Alpha and also Omega, in the Lancet, Courier, and other periodicals. The author is a physician of long experience and ample observation.*

---

25. Observations on the Medical Treatment of Insanity. By ED. G. SEYMOUR, M.D. Physician to St. George's Hospital, &c. 8vo, pp. 95. Longman and Co. 1832.

---

26. A Critical and Experimental Essay on the Circulation of the Blood; especially as observed in the Minute and Capillary Vessels of the Batrachia and of Fishes. By MARSHALL HALL, M.D. &c. 8vo, pp. 187, with ten plates. Seeley, 1832.

---

27. A Brief Description of Surgical Apparatus; intended to accompany a Series of Delineations of the most important Auxiliaries of Surgery. By HENRY T. CHAPMAN, M.R.C.S. late House Surgeon to St. Bartholomew's Hospital. 8vo, pp. 115. London, 1832.

---

28. Official Reports made to Government, by Drs. RUSSELL and BARRY, on the Disease called Cholera Spasmodica, as observed by them during their Mission to Russia in 1831; with an Appendix and other Papers, &c. 8vo, pp. 147. February, 1832.

---

29. A Treatise on Retention of Urine, caused by Strictures in the Urethra; and of the Means by which Obstructions of this Canal may be effectually removed. By THEODORE DUCAMP, Doctor of Medicine of the Faculty of Paris, &c. translated from the French, with Notes and Additions. By WILLIAM M. HERBERT, M.D. 8vo, pp. 219, four plates. New York, 1827.

☞ *We shall take some notice of this volume.*

---

30. A Treatise on Surgical Anatomy; on the Anatomy of Regions, considered in all its relations with Surgery. Illustrated by plates, representing the principal regions of the body. By A. L. M. VELPEAU, M.D. P. Agiegè Stagiare to the Faculty of Medicine of Paris, &c. In two volumes, Translated from the French, with additional notes. By JOHN W. STERLING, M.D. M.R.C.S.L. &c. Vols. II. 8vo, pp. 456—523. Plates. New York, 1830.

---

31. An Account of the Beulah Saline Spa at Norwood, in Surrey; containing a description of its Medicinal Properties and Effects; of the diseases in which it is remedial, &c. By GEORGE HUME WEATHERHEAD, M.D. 8vo, pp. 39. 1832.

---

32. The Laws and Progress of the Epidemic Cholera; illustrated by Facts and Observations. By THOMAS HANCOCK, M.D. 8vo, pp. 118. February, 1832.

*⁂ *We shall notice this work anon.*

---

33. The Principles and Practice of Obstetric Medicine, &c. By DAVID D. DAVIS, M.D. Professor of Midwifery in the University of London. Part V. Price 2s.

☞ *We shall notice this and the succeeding parts as they issue from the press.*

---

34. Dr. Townsend's Chart of the Physical Signs of Diseases of the Lungs. Price 3s.

☞ *A very useful chart for the young auscultator.*

---

35. Practical Observations on the New System of Warming Dwelling-houses, Cathedrals, Churches, Theatres and other Public Buildings, with Hot Water, &c. By HENRY W. DEWHURST, Esq. Duodecimo, pp. 37. 1832.

☞ *This contains much useful information for all concerned in the above subject.*

36. The Dublin Journal of Medical and Chemical Science; exhibiting a Comprehensive View of the latest Discoveries in Medicine, Surgery, Chemistry, and the Collateral Sciences. 8vo, pp. 116. Hodges and Smith, Dublin, March, 1832, price 3s. 6d.

---

37. A brief Statement of the Advantages and Safety of Fumigating Baths, &c. By J. GREEN, Surgeon.

---

38. Dissertations on Malaria, Contagion, and Cholera; explaining the Principles which regulate Epidemic, Endemic, and Contagious Diseases, with a View to their Prevention, &c. intended as a Guide to Magistrates, Clergymen, &c. By W. AITON, M.D. 8vo, pp. 72—220. March, 1832.

---

39. Hints relating to the Cholera in London. By THOMAS HODGKIN, M.D. &c. &c.

☞ *This is in the form of a letter. It contains many judicious suggestions, ingenious reflections, good feeling, and good sense. Dr. H. objects to the formation of cholera hospitals. In this city, he thinks, they are worse than useless. Not one hundredth part of the patients can possibly be received into them. Dr. H. regrets that cholera patients have been refused admission into the common hospitals; so do we. Separate wards and separate nurses might have been provided for them, if such were considered necessary. We have only to look into the daily papers for instances of the shocking, monstrous, and unchristian effects of the doctrines of the contagionists and alarmists. Dr. Hodgkin deserves well of the public.*

---

40. Introductory Lecture to a Course of Forensic Medicine, delivered in the Anatomical Theatre of St. Thomas's Hospital, November, 1831. By GEORGE BURROWS, M.D. &c. 8vo, stitched, pp. 32. 1832.

☞ *A very able discourse; not less distinguished for the erudition than for the high moral and sound political feeling of the author.*

---

41. Lectures delivered at the Mechanics Institution, 19th Dec. 1831, and 13th Feb. 1832, on Oxygen, Carbon, and Vitality, the three great Agents in the

Physical Character of Man. With remarks on Asiatic Cholera. By GEORGE REES, M.D. &c. 8vo, stitched, pp. 107. London, 1832.

☞ *Very instructive and interesting Lectures. Some of the passages do honour to the heart of the Lecturer, and are but too applicable to the present times of luxury for an oligarchy, misery for millions.*

---

42. History and Medical Treatment of Cholera, as it appeared in Sunderland in 1831, illustrated by numerous Cases and Dissections. By W. HASELWOOD, M.D. and W. MORDEY, Surgeon, in charge of the Cholera Hospital in that Town.

☞ *Too late for notice in the present number.*

---

43. Thoughts on Cholera Asphyxia. By ROBERT BREE, M.D. F.R.S. &c. 8vo, stitched, pp. 71. London, 1832.

☞ *Very ingenious, but the author has never seen the disease.*

---

44. On the Epidemic Cholera, and other prevalent Diseases of India. By SAMUEL DICKSON, Assistant Surgeon to the Royals, lately on the Madras Establishment. 8vo, pp. 143. Edinburgh and London, 1832.

☞ *We may possibly take notice of this in our next.*

---

45. Anatomical Demonstrations; or Colossal Illustrations of Human Anatomy. By PROFESSOR LEERIG. Translated from the German. Part 2. London. Published by A. Schloss, 103, St. Martin's Lane, Charing Cross. 1831. Price 8s. 6d. each part, or 12s. coloured

☞ *The present Part contains five large lithographed plates, representing the base of the brain and cranium, with the nerves, the origin of the optic, and the continuation of the white matter of cerebrum, cerebellum, and chord, illustrated by a vertical and horizontal section. This part equally deserves encomium with the former, which we noticed in a preceding number of the Journal. M. Schloss deserves well of the professional public, for his spirited attempt to import the minute anatomy of Germany into England. We shall continue to notice this series of anatomical plates as they successively issue from the press.*

*We will here avail ourselves of the opportunity of noticing the magnificent work of Mr. Swan upon the Nerves. It is indeed magnificent, and calculated to confer honour not merely on the individual, but on the anatomical character of the profession of Great Britain. If that work is not patronized by our public bodies, and by those private persons to whom fortune has been bounteous, then shame, we say, on one and all. The Medical Corporations must assist the author by purchasing a certain number of impressions. If they do not they will add one more damning blot to escutcheons, already far from too fair. It is but justice to the College of Surgeons to acquit them of illiberality on this occasion.*

*We had intended to have made more particular remarks on Mr. Swan's work. We regret that we have been prevented from doing so from having unfortunately mislaid it. In our next number we hope to be enabled to redeem our pledge.*

### INDEX.

A.

Abdomen, on exploration of ...... 446
Abortion, Dr. Lee on ............ 446
Abscess of the brain............. 255
Abscess of the lung, Dr. Tweedie on, 449
Abscess external to the larynx, cases of......................... 591
Abstinence, Dr. Hall on .......... 450
Achor and acne, Dr. Todd on...... 452
Acne, M. Biett on................ 396
Acupuncture, Dr. Elliotson on .... 453
Africa, Mr. Boyle on the diseases of, 408
African fever, cases of........... 417
Age, Dr. Roget on ............... 453
Ainslie, Dr. on cholera........... 439
Air, Dr. Clark on change of ...... 456
Albers, Dr. on cholera in Moscow.. 170
Alopecia, Dr. Todd on............ 456
Alteratives, Dr. Conolly on. ...... 457
Amenorrhœa, Dr. Locock on...... 458
Amputation, immediate, M. Larrey on ........................... 60
Amputations, illustrations of...... 242
Analysis, Dr. Todd's book of...... 36
Anatomy, Massachusetts report on, 101
Anatomy, surgical, M. Velpeau's .. 478
Anatomy, lectures on ............. 239
Andral, M. on hyperæmia ........ 87
Aneurism, iliac, successfully treated —cases ......................... 514
Aneurism, carotid, operation for .. 520
Aneurism, popliteal, operation for.. 524
Angina pectoris, Dr. Forbes on .... 459
Animalculæ in the air, producing cholera ......................... 182
Animalculæ preceding cholera .... 184
Animalcular epidemic, in 1346 .... 183
Anodynes, Dr. Whiting on........ 461
Anthelmintics, Dr. Thomson on .. 462
Aortic aneurism, Dr. Hope on .... 463
Aphonia—aphthæ, Dr. Robertson on ........................... 466
Apoplexy, Dr. Bright on.......... 12
Apoplexy of the spinal cord ....... 19
Apoplexy, blood-letting in ........ 19
Apoplexy, diuretics in ............ 21
Apoplexy, serous and nervous .... 228
Apoplexy, Dr. Clutterbuck on ..... 467
Apoplexy, pulmonary, Dr. Townsend on ............................ 470
Asthma and auscultation, Dr. Forbes on ............................ 475
Atmospheric influence on cholera.. 202
Auscultation applied to the heart .. 360

B.

Baths of iodine in scrophula ...... 115
Beaumont, Mr. on fractures ...... 247
Bell, Mr. on cholera............... 186
Bell, Mr. his letter to Sir H. Halford 224
Beulah Spa, account of............ 554
Biett, M. his lectures, by Cazenave, 382
Bladder, wound of the............ 59
Bladder, on the form and disposition of the ......................... 144
Bladder, states of, *quoad* lithotrity, 152
Blood, microscopic researches on the 256
Blow-pipe, use of the ............ 381
Blue skin in malformed hearts .... 73
Boyle, Mr. on the diseases of Africa, 408
Brain, tumours in the ............ 22
Brain, hypertrophy of the ........ 26
Brain, membranous alterations of.. 28
Brain, cases of mollescence of .... 228
Brain, encysted abscess of ........ 255
Brain, on the functions of the .... 423
Brain, size of, its influence........ 427
Brain, diseased in insanity........ 427
Brain, ulceration of the .......... 601
Brain, inflammation of the........ 598
Bright, Dr. his medical cases...... 1
Bright, Dr. his medical reports .... 321
Brown, Dr. his letter to Dr. Johnson 301
Brown, Mr. on cholera ........... 584
Burns and scalds, M. Perry on .... 257
Burns, cases of.................. 259
Burns, our treatment of.......... 261

C.

Calculi, vesical, forms of.......... 146
Calomel, effects of mixture with bile, &c........................... 611
Cameron, Mr. his letter on cholera, 633
Cancer of the stomach mistaken ... 231
Carotid aneurism, operation for .... 520
Cazenave on diseases of the skin .. 382
Celsus, Dr. Collier's.............. 156
Celsus, Mr. Lee's ................ 157
Celsus, was he a practitioner?...... 159
Cerebral pressure, Dr. Bright on ... 2
Cerebral effusion and diseased kidney 5
Cerebral pressure from injury .... 33
Cerebritis, Dr. Crawford on ...... 599
Charcoal, suffocation from the fumes of ............................ 4
Chemistry, Dr. Reid's elements of.. 374
Children, partial fractures in...... 588
Cholera at Port Glasgow.......... 130
Cholera from fruit ............... 133

Cholera, the epidemic .............. 163
Cholera, Prof. Lichtenstadt on.... 164
Cholera at Orenburg in Aug. 1829.. 164
Cholera, convulsions after death in, 165
Cholera, not *imported* into Orenburg 165
Cholera, Sir William Crichton on .. 168
Cholera, its introduction into Russia, 168
Cholera, causes and prevention of.. 169
Cholera in Moscow .............. 170
Cholera, Moscow report on the .... 172
Cholera, Dr. Walker's letter on.... 175
Cholera, Sir W. Pym's letter on.... 177
Cholera, Sir H. Halford's first letter on ........................ 178
Cholera, Sir Henry's second letter on 179
Cholera, Mr. Kennedy on .......... 180
Cholera, Mr. Neale on............ 181
Cholera, Mr. Bell on.............. 186
Cholera, bleeding in .............. 187
Cholera, affection of the ganglionic system in .................... 188
Cholera, mode of propagation of .. 189
Cholera, singularities of .......... 190
Cholera, stated to accompany troops 191
Cholera, mischiefs of restrictive cordons in ...................... 192
Cholera, venæsection in .......... 192
Cholera, Mr. Orton on............ 193
Cholera, symptoms of ............ 194
Cholera, post-mortem appearances, 196
Cholera, proximate cause of ...... 197
Cholera, compared with other epidemics ...................... 198
Cholera, contagiousness of........ 199
Cholera, atmospheric influence in.. 202
Cholera, influence of locality in.... 205
Cholera along rivers, considered ... 206
Cholera, susceptibility of mankind to 208
Cholera, treatment of ............ 209
Cholera, Mr. Searle on............ 211
Cholera, cause and treatment of ... 211
Cholera, Dr. Lefevre on .......... 213
Cholera at St. Petersburgh........ 214
Cholera, symptoms of............ 215
Cholera, primary fatality of........ 216
Cholera, treatment of ............ 216
Cholera, Dr. Young on ............ 217
Cholera, its progress ............ 218
Cholera, origin of at many places .. 219
Cholera, non-contagiousness of .... 220
Cholera, miscellaneous works on .. 221
Cholera in England .............. 266
Cholera, ultra and moderate Boards 267
Cholera, Dr. Johnson's opinions on, 268
Cholera as contagious as typhus.... 268
Cholera in Newcastle and Sunderland 269
Cholera constitution of 1831 ...... 270
Cholera in St. James's workhouse .. 271
Cholera in Piccadilly ............ 272
Cholera, is it a new disease? ...... 273
Cholera, reputed peculiarities of.... 273
Cholera, Dr. Johnson's propositions on ........................ 274
Cholera, Drs. Russell and Barry on, 277
Cholera in Sunderland, Dr. Tinn on, 280
Cholera, how propagated ........ 281
Cholera, a *Chinese* blessing........ 283
Cholera, Dr. Sanders' letter on.... 297
Cholera, Dr. Brown's letter on .... 301
Cholera, Dr. Negri on ............ 303
Cholera as it is, our views of ...... 304
Cholera at Danzig, Dr. Hamett on, 305
Cholera, its origin in that city ..... 305
Cholera, its prevalence there...... 307
Cholera, its symptoms there ...... 308
Cholera, its pathologic characters there ........................ 310
Cholera, treatment of there ...... 313
Cholera, general treatment of...... 315
Cholera, contagion of, considered by facts ........................ 318
Cholera, Mr. Bell on, 2d edition.... 434
Cholera at Haddington .......... 435
Cholera at Newton .............. 437
Cholera, Dr. Ainslie on .......... 439
Cholera, Mr. Thackrah on ........ 441
Cholera at Leeds; remarkable .... 442
Cholera, on the etiology of the .... 484
Cholera, Orton's theory of ........ 485
Cholera, Russian embassy on...... 489
Cholera, O'Shaughnessy and Clanny on ........................ 496
Cholera, report of the Madras Board on ........................ 497
Cholera at Arcot, in 1787 ........ 499
Cholera described by Bontius...... 499
Cholera described by Dr. Paisley, in 1774 ........................ 501
Cholera described by Sonnerat in 1774 ........................ 501
Cholera at the Mauritius.......... 502
Cholera at Ganjam in 1781........ 503
Cholera described by Curtis in 1782, 503
Cholera described by Girdlestone .. 504
Cholera described by Dr. Duffin in 1787 ........................ 505
Cholera described by Dr. Davis in 1787 ........................ 506
Cholera described by Mr. Thompson 507
Cholera in the Northern Circars, in 1790 ........................ 507
Cholera since 1787 .............. 508
Cholera in 1814.................. 509
Cholera in Travancore............ 510
Cholera, names of................ 512
Cholera at Sunderland, Dr. Ogden on ........................ 533
Cholera at Sunderland, &c. Dr. Lawrie on .................... 541
Cholera at Gateshead, Dr. White on, 541

Cholera at Kirkintilloch .......... 542
Cholera hospitals, Dr. Lawrie on .. 544
Cholera, endemic character of .... 546
Cholera, predisposition to ........ 546
Cholera, symptoms of ............ 548
Cholera, treatment of ............ 550
Cholera at Sunderland, Mr. Greenhow on ...................... 555
Cholera; the ultra-contagionists .. 556
Cholera; analysis of its symptoms.. 567
Cholera, treatment of............ 558
Cholera, causes of................ 561
Cholera, influence of fear in causing, 562
Cholera, inefficacy of quarantine against ..................... 564
Cholera, arguments for contagion of, 566
Cholera hospitals, mortality of .... 570
Cholera at Musselburgh, Mr. Moir on 572
Cholera; Edinburgh contagionists, 572
Cholera, erratic nature of ........ 575
Cholera, Mr. Dodd on............ 576
Cholera, Dr. Fergusson on ........ 580
Cholera; effects of fear .......... 583
Cholera, Mr. Brown on .......... 584
Cholera, fumigation in .......... 586
Cholera and cheap whiskey........ 587
Cholera, Dr. Clarke's case of...... 605
Cholera, Dr. O'Halloran on ....... 608
Cholera, description of, by the Madras Board.................... 609
Cholera, varieties in.............. 610
Cholera; particular symptoms considered ....................... 613
Cholera, rapid recovery from, in Madras......................... 616
Cholera, state of blood in ........ 617
Cholera, terminations of, in Madras, 618
Cholera, morbid appearances of.... 620
Cholera, tabular analysis of the individual Madras reports on........ 622
Cholera, Editor's remarks on...... 627
Cholera, consecutive fever in Asia and Europe .................. 629
Cholera described by former authors, 631
Cholera at Haddington, Drs. Lorimer and Burton on ................ 640
Cholera, sporadic, in many villages, 643
Cholera, treatment of at Haddington 644
Cholera, progress of, in England .. 645
Cholera at Sunderland............ 645
Cholera at Newcastle ............ 647
Cholera, North and South Shields, 648
Cholera, Gateshead .............. 649
Cholera, Haddington ............ 650
Cholera, Tranent ................ 651
Cholera, Preston Pans ............ 652
Cholera, Edinburgh.............. 652
Cholera, propagation by contagion, 653
Cholera, symptoms of............ 655
Cholera; premonitory diarrhœa.... 656
Cholera, cases of ................ 657
Cholera, cadaveric appearances in.. 660
Cholera; ratio signorum ......... 665
Cholera, treatment of............ 669
Cholera, causes of .............. 672
Cholera, Dr. Yates' letter on...... 674
Cholera, concluding remarks on .. 675
Cholera, address to the profession, on a cholera association ........ 676
Chorea, Dr. Bright on............ 328
Clark, Dr. on change of air........ 456
Classifications of diseases.......... 382
Combe, Dr. on mental derangement 423
Congenital malformations of the heart .......................... 68
Convalescent retreats for the poor.. 82
Convulsions, Dr. Bright on........ 343
Cooper, Mr. on anatomy.......... 239
Cooper, Mr. Bransby, his ligature of the iliac...................... 514
Corrigan, Dr. on dothinenterite.... 227
Cruveilhier, M. on laryngitis ...... 591
Cyclopædia of Medicine, Part I..... 445
Cyclopædia of practical medicine, Part III...................... 598

D.

Dantzig, cholera at.............. 305
Davis, Dr. on obstetric medicine .. 235
Davis, Dr. on obstetric medicine .. 579
Delpech, M. on varicocele ........ 252
Diabetes, Dr. Bright on .......... 9
Diseases of the heart, Dr. Hope on, 262
Diseases of the heart, their importance ...................... 263
Dispensary patients, a picture of .. 81
Dissection of the dead, report on .. 101
Dobson, Mr. on the epidemic cholera at Leeds.................. 636
Dodd, Mr. on cholera ............ 576
Dothinenterite, Dr. Corrigan on .. 227

E.

Eczema, M. Biett on ............ 386
Eczema, treatment of............ 390
Elements of surgery, Mr. Liston's.. 242
Elephantiasis, M. Biett on ........ 406
England, cholera in.............. 266
England, history of cholera in .... 645
English manufacturers, health of .. 77
Epidemic cholera................ 163
Epidemic, present, etiology of the.. 484
Epidemics, causes of ............ 199
Epilepsy, Dr. Bright on .......... 336
Epilepsy combined with apoplexy .. 340
Erysipelas, M. Biett on .......... 385

F.

Facts, Dr. Todd's method of classifying ........................ 40

Fergusson, Dr. on cholera ........ 580

Fever, climatorial and endemic, of Africa.......................... 411

Fractures, treated by plaster casts, 247

Fractures, partial, in children...... 588

Frank's description of cholera..... 631

Fungoid tumour of neck—operation fatal.......................... 525

Fungus cerebri, cases of.......... 518

G.

Gateshead, Dr. White on cholera at, 541

Greatrex, Mr. his letter on Dr. Stevens...................... 295

Greenhow, Mr. on cholera........ 555

Gregory's Conspectus, selections from.......................... 513

Gunshot wounds, enlargement of.. 50

Guthrie, Mr. his ligature of the iliac, 516

H.

Hacket, Dr. his letter to Dr. Johnson, 289

Haddington, cholera at .......... 640

Haddington, non-contagiousness of cholera...................... 641

Halford, Sir H. his first letter on cholera....................... 178

Halford, Sir H. his 2d letter on ditto 179

Hall, Dr. his experiments on loss of blood......................... 364

Hamett's valuable report on Cholera 305

Hanging, effects of, on the brain .. 3

Headachs, Dr. Vaughan on........ 83

Headachs, divisions of............ 85

Heart, congenital malformations of, 68

Heart, symptoms of malformations of the.......................... 71

Heart, relative anatomy of the .... 265

Heart, Dr. Hope on the action of the 358

Heart, sounds of the, in disease.... 362

Heart, on murmurs of the ........ 364

Heart, inflammation of the........ 366

Hemicrania, M. Piorry on ........ 236

Hemicrania, Dr. Bright on........ 334

Hernia cerebri, cases of .......... 518

Herpes, M. Biett on.............. 392

Heurteloup, Baron, on lithotrity .. 134

Heurteloup, M. his instruments .. 140

Hope, Dr. on diseases of the heart, 262

Hope, Dr. on diseases of the heart.. 356

Hope, Dr. on aortic aneurism .... 463

Hydrocyanic acid, properties of.... 378

Hydrophobia, Dr. Bright on ...... 349

Hyperæmia, M. Andral on ........ 87

Hyperæmia, active or sthenic ..... 88

Hyperæmia, secondary, characters of 89

Hyperæmia, bloodletting and emetic tartar in .................. 91

Hyperæmia, state of the capillaries in.......................... 92

Hyperæmia, asthenic ............ 94

Hyperæmia, mechanical.......... 97

Hyperæmia, cadaveric............ 98

Hyperæmiæ, co-existence of several, 91

Hysteria, Dr. Bright on .......... 323

Hysteria, aping various affections .. 324

I.

Iliac fossa, phlegmonous tumours in, 225
Iliac aneurism, successfully treated, cases ........................ 514
Illustrations of amputations ...... 242
Imperforate vagina, operation for .. 602
Impetigo, M. Biett on............ 396
Insanity, post-mortem appearances of........................... 538
Iodine, M. Lugol on, in scrofula.... 109
Iodine, effects of ................ 111
Iodine, M. Lugol's form of........ 112
Iodine, external local effects of.... 113
Iodine, internal..................
Iodine for scrofulous tubercles .... 119
Iodine for scrofulous ophthalmia .. 120
Iodine for scrofulous cutaneous tissue 121
Iodine for scrofulous cellular tissue, 123
Iodine for scrofulous bones........ 123
Iodine, preparations of............ 125
Iodine, caustic .................. 127
Ioduret of lead, effects of.......... 127
Ioduret of mercury .............. 129
Iodurettted baths in scrofula ...... 115
Irritation ; what is it? ........... 321

J.

Johnson, Dr. his propositions on cholera ...................... 274
Johnson, Dr. Hackett's letter to .. 289
July, 1830, M. Larrey on the wounded of ....................... 49
July, 1830, kinds of wounds received in ..................... 50

K.

Kennedy, Mr. his impartiality .... 180
Kidney, ruptured, case of ........ 594

L.

Lactation, remarks on............ 154
Laidlaw, Mr. his surgical report.... 594
Larrey, M. Hippolite, on the wounded of July .................. 49
Laryngitis, M. Cruveilhier on ..... 591
Lawrie, Dr. on cholera at Sunderland ........................ 541
Lead, palsy from ................ 29
Lectures on anatomy ............ 239
Lee, Dr. on abortion............. 446
Leeds, epidemic cholera at, in 1825, 636
Lefevre, Dr. on cholera .......... 213
Lepra, M. Biett on .............. 402
Lichen, M. Biett on.............. 402
Lichtenstadt, Dr. on cholera ...... 164

Liston, Mr. on surgery ........ .. 242
Lithotritic instruments, considerations on .................... 148
Lithotritist, picture of a .......... 153
Lithotrity, Heurteloup on ........ 134
Lithotrity and lithotomy, parallel between .................... 138
Lithotrity, history of ............ 139
Lithotrity, circumstances favourable to, &c...................... 150
Locality, influence of on cholera.... 205
Lugol, M. on scrofula ............ 109
Lung, Dr. Tweedie on abscess of .. 449

M.

Macrobin, Dr. on insanity ........ 538
Madras Board, report of the, on cholera ...................... 497
Malaria, Volta's experiments on.... 490
Manchester, numbers of the sick of, 78
Manufacturing poor, Mr. Roberton on the........................ 77
Marshall Hall, Dr. on abstinence .. 450
Marshall, Mr. his cases of Glasgow, 130
Massachusetts' report on anatomy.. 106
Massachusetts' old laws of, on anatomy 104
Massachusetts' new act on anatomy, 106
Medical cases, Dr. Bright's ........ 1
Medical reports, Dr. Bright's...... 321
Medicine, Cyclopædia of, Part I. ... 445
Medicine, how a science of experience .......................... 38
Meningitis, Dr. Quain on ........ 598
Mental derangement, Dr. Combe on 423
Mental derangement, always from cerebral disease................ 427
Mercury, paralysis from .......... 32
Mercury, delicate tests of ........ 379
Microscopic researches on the blood, 256
Moir, Mr. on cholera at Musselburgh 572
Mollescence of brain, cases of ..... 228
Morbid anatomy, considerations on, 46
Morton, Dr. on lactation........... 154
Morton on choleric fever in his time, 303
Moscow, cholera in ............... 171
Moscow, report of the council of, on cholera ...................... 172
Musselburgh, cholera at ........... 585

N.

Neale, Mr. on cholera ............ 181
Needle swallowed, and taken from bladder ........................ 251
Neuralgia, Dr. Bright on.......... 332
Nitric acid in tooth-ache.......... 578
Nitrous oxyde gas, properties of the, 377

O.

Obstetric medicine, Dr. Davis on .. 235
Obstetric medicine, Dr. Davis on .. 579
Ogden, Dr. on cholera at Sunderland 533
O'Halloran's letter on cholera ..... 608
Operatives, miseries and vices of .. 80
Orton, Mr. on cholera............. 193
Orton, Mr. his theory of cholera.... 486
O'Shaughnessy, Dr. his translation of Lugol .......................... 109
O'Shaughnessy's analysis of cholera secretions ...................... 496
Oxalic acid, distinguished from Epsom salts ...................... 379

P.

Paget, Dr. on malformations of the heart ........................ 68
Paralysis, aped by hysteria ........ 326
Paralysis from lead ................ 29
Paralysis from mercury ........... 32
Parotid tumour, extirpation of a ... 603
Pemphigus, M. Biett on ........... 394
Pericarditis, Dr. Hope on ......... 366
Perry, Mr. on burns and scalds.... 257
Pharmacopœia, introduction to the, 513
Phlegmonous tumours in right iliac fossa ........................ 225
Piorry, M. on syncope...... : .... 233
Piorry on hemicrania ............. 236
Plaster of Paris casts for fractures.. 247
Porrigo, M. Biett on............... 398
Port Glasgow, cholera at........... 130
Prolapsus of rectum, Mr. Salmon on 369
Prolapsus of rectum, treatment of .. 371
Pruritus of the genitals, Dr. Davis on 579
Psoriasis, M. Biett on ............ 405
Pulmonary apoplexy, Dr. Townsend on ........................... 470
Pym, Sir W. his letter on cholera.. 177

R.

Rectum, Salmon on prolapsus of .. 369
Rectum, ulcer of, cured by bougie.. 256
Reid, Dr. his elements of chemistry, 374
Remittent fever of Africa ......... 411
Revolution of July, M. Larrey on the 49
Roberton, Mr. on the health of manufacturers ........ ......... 77
Rupia, M. Biett on ............. 395
Russell and Barry, Drs. on cholera, 277
Russian Embassy on cholera ...... 489

S.

Salmon Mr. on prolapsus recti .... 369
Sanders, Dr. his letter to Sir H. Halford .. : ................... 297
Scabies, M. Biett on............... 392
Scrofula, M. Lugol on iodine in.... 109
Scrofula treated by iodine ........ 117
Scrofulous tubercles.............. 119
Scrofulous ophthalmia, iodine in ... 120
Scrofulous eruptions............... 121

Searle, Mr. on cholera............ 211
Seeds, Dr. his remedy for ophthalmia 607
Sierra Leone, Mr. Boyle on........ 409
Skin, Cazenave on diseases of the.. 382
Smyrna, algid fever of............ 607
Soap cerate, Mr. Fergusson on .... 590
Spa, Beulah, account of .......... 554
St. Petersburgh, cholera at........ 214
Stevens, Dr. criticised by Dr. Hackett, 289
Stomach, cancer of, mistaken...... 231
Suicidal wound of throat, fatal .... 596
Sunderland, cholera in ........... 280
Sunderland, Dr. Ogden on cholera at 533
Surgery; Mr. Liston on .......... 242
Surgical anatomy, M. Velpeau's.... 478
Syncope and cerebral congestion .. 233

T.

Teaching, Dr. Todd's method of.... 45
Terrene exhalations, as causes of cholera ..................... 166
Tetanus, Dr. Bright on .......... 343
Thackrah, Mr. on cholera ........ 441
Throat, Velpeau's description of the, 481
Throat, fatal suicidal wound of .... 596
Todd, Dr. his book of analysis...... 36
Todd, Dr. his analytical scheme ... 42
Toothache, nitric acid in.......... 578
Topographical facts on cholera .... 489
Torti on cholera fever in 1700.... 303
Townsend, Dr. on pulmonary apoplexy ..................... 470
Tumour, fungoid, of neck, fatal case of........................ 525
Tumour, parotid, extirpation of a.. 603
Tumours in the brain, cases of .... 22

U.

Ulcer of rectum cured by bougie ... 256
Urethra, with a reference to lithotrity......................... 141
Uterus to be attended to in hysteria, 328

V.

Vagina, imperforate, operation for, 602
Valvular disease of heart, sounds in, 363
Varicocele, M. Delpech on ........ 252
Varicocele, veins tied for ......... 252
Varicocele, death from operation... 253
Varicocele, cures of .............. 254
Vaughan, Dr. on headaches........ 83
Velpeau, M. his surgical anatomy .. 478
Volta's experiments on malaria.... 490

W.

Walker, Dr. on cholera in Moscow, 175
White swelling, iodine in ......... 124
White, Dr. on cholera at Gateshead, 541
Wounds, M. Larrey's treatment of, 51
Wounds of the head, M. Larrey on, 52
Wounds of the face .............. 54
Wounds of the eyes .............. 55
Wounds of the neck .............. 56
Wounds of the chest.............. 57
Wounds of the abdomen .......... 58
Wounds of the extremities ........ 60
Wounds of the thigh.............. 64

Y.

Yates, Dr. his letter on cholera.... 606
Young, Dr. on cholera in India .... 218

